# Integrated Human Exposure to Air Pollution

**DOI:** 10.3390/ijerph18052233

**Published:** 2021-02-24

**Authors:** Nuno Canha, Evangelia Diapouli, Susana Marta Almeida

**Affiliations:** 1 Centro de Ciências e Tecnologias Nucleares (C^2^TN), Instituto Superior Técnico, Universidade de Lisboa, Estrada Nacional 10, Km 139.7, 2695-066 Bobadela LRS, Portugal; smarta@ctn.tecnico.ulisboa.pt; 2 Centre for Environmental and Marine Studies (CESAM), Department of Environment and Planning, University of Aveiro, 3810-193 Aveiro, Portugal; 3 National Centre for Scientific Research “Demokritos”, Agia Paraskevi, 15341 Athens, Greece; ldiapouli@ipta.demokritos.gr

Air pollution is one of the major environmental health problems that people face nowadays, affecting everyone in the world. The World Health Organization has estimated that, in 2016, ambient air pollution caused 4.2 million premature deaths worldwide, in both cities and rural areas, while household air pollution caused 3.8 million deaths, mainly in low and middle-income countries [1].

Usually, citizen exposure to air pollutants is calculated based only on concentrations of pollutants monitored using air quality monitoring stations from environment national agencies. These monitoring stations focus on outdoor air quality and, most of them, are located in urban centres. However, this approach fails to account for all components of exposure since:

- There is a high variability of air pollutant concentrations within a city, depending on the city topography and the existence (or not) of specific pollution sources (such high traffic areas);
- The time–activity patterns and the use of spaces are very heterogeneous within the population;
- People spend around 90% of their time in indoor environments;

Therefore, human exposure during a full day cannot be reflected only by outdoor exposure and should consider all micro-environments where individuals spend their time (e.g., home, workplace, transports, leisure, and others) and the time spent in them.

Moreover, the characterization of air pollutants in indoor and outdoor environments is essential to understand the integrated human exposure to them and, thus, to improve those exposure assessments.

The Special Issue “Integrated human exposure to air pollution” aimed to increase the knowledge about human exposure in different micro-environments or when citizens are performing specific tasks, to demonstrate methodologies for the understanding of pollution sources and their impact on indoor and ambient air quality, and, ultimately, to identify the most effective mitigation measures to decrease human exposure and protect public health. Taking advantage on the latest available tools, such as internet of things (IoT), low-cost sensors and a wide access to online platforms and apps by the citizens, new methodologies and approaches can be implemented to understand which factors can influence human exposure to air pollution. This knowledge made available to the citizens, along with the awareness of the impact of air pollution on human life and earth systems, can empower them to act, individually or collectively, to promote behavior changes aiming to reduce pollutants’ emissions.

Overall, this Special Issue gathers fourteen peer-reviewed and open access articles that provide new insights regarding these important topics within the scope of human exposure to air pollution. A total of five main areas were discussed and explored within this special issue, which are described below, and, hopefully, can contribute to the advance of knowledge in this field.

## 1. New Methodologies to Assess Human Exposure

In recent years, the use of low-cost sensors for assessing environmental parameters was a landmark regarding personal exposure assessments due to their portability, low price and easy handling, allowing to add multi sensors to monitor different parameters, along with global positioning system (GPS) sensor and transmission of acquired data to online platforms. Such combination allows to gather different types of information/data in cloud environments, usually in a great amount, which contributed to the concept of big data in the field of human exposure assessments regarding air pollution. However, a critical issue on the use of such type of monitoring devices is their validation towards reference methods to assure the reliability of results.

A study conducted in Texas (USA) [2] focused on this issue where low cost particle sensors (targeting suspended particulate matter) were developed, calibrated and tested in real life conditions. The developed monitoring unit used an Arduino interface, a commercially available dust sensor (namely, Sharp GP2Y1010AU0F), a Global Positioning System (GPS) sensor (to provide the location of the measurements) and a communications module to transmit data to an online platform. The authors also provided a historical and critical overview of the evolution of these types of low-cost sensors and their performance, based on a literature review. The dust sensors employed in the monitoring units were calibrated regarding a reference aerosol monitor, using a mixing chamber and generation of sodium chloride particles, where the sensors showed standard deviations for replicate measurements of 3-6 µg/m^3^. To show the applicability of the developed set-up, measurements in real life conditions were conducted both at indoor and outdoor environments in Lubbcok, Texas. Analysis of the results allowed to identify, for instance, increased exposure levels to particulate matter nearby restaurants and when cooking at home. The gathered data also allowed to build maps with spatial location of the monitored PM levels.

Using a case study in Madrid (Spain), a new methodology, based on the Internet of Things (IoT) approach and using low cost sensors of air pollutants (PM_2.5_, CO_2_ and VOC) along with available outdoor air quality data, was proposed for estimating personal air pollution exposure (PAPE) [3]. Individual’s PAPE was estimated based on combined outdoor and indoor data with spatio-temporal activity patterns. The understanding of instantaneous PAPE, by means of a forecasting approach, may be useful for individual awareness, as for example, to decide travel routes in order to minimize their exposure, as it was shown in this case study. Overall, this study provided a new framework for an Air Quality Decision Support System (AQDSS), taking advantage of the nowadays’ technologies and showing its applicability.

## 2. Citizens Empowerment Regarding Air Pollution

The importance of public awareness on air pollution issues and its impacts was highlighted in a study conducted in Shanghai (China), where the relationship between air pollution levels and the expression of concern of the city’s residents was evaluated [4]. Using information of a period of two thousand days (with a daily frequency) (from 2013 to 2019) regarding the air quality index provided by the local environmental monitoring stations and an index about the internet searches of the residents based in selected keywords, an empirical analysis was conducted using a vector autoregression model.

Using this strategy, three main findings were reported about the awareness levels of citizens regarding this topic: (1) degradation of air quality was perceived by citizens within one day; (2) information regarding air quality issues in another major city (Beijing) conducted to a concern on the inhabitants of Shanghai about local air quality; and (3) higher concern levels of the citizens had a beneficial impact on local air quality, which can be explained by their environmental awareness that promoted pro-environmental behaviors, along with public pressure toward governmental actions to tackle air pollution. This study highlights the importance of knowledge transfer about air pollution and their impacts to the citizens, which is reflected by a higher awareness level that may lead to actions to improve air quality and, consequently, to minimize citizens’ exposure to air pollutants and its negative impacts on human health.

## 3. Outdoor Pollution Sources

Identifying pollution sources is crucial to define mitigation measures to improve air quality at specific locations. In urban areas, one of the main pollution sources is traffic and several cities worldwide are implementing traffic restrictions taking into account the vehicles category and their pollution potential. One example is the multi-year progressive traffic policy implemented in an extended limited traffic zone of the municipality of Milan (Italy) that started in the beginning of 2019, with the restriction of circulation of some classes of highly polluting vehicles (Euro 0 petrol vehicles and Euro 0 to 3 diesel vehicles). Another important step in these processes is a continuous evaluation of those measures in order to improve them or change them according to the desired results. For this reason, monitoring data is essential along with statistical tools which can provide information regarding trends and forecast outcomes. Such type of analysis was conducted for the Milan case [5], where the early-stage impact of this policy on two specific vehicle-generated pollutants (namely, total nitrogen oxides (NO_x_) and nitrogen dioxide (NO_2_)) was evaluated.

For this, daily environmental data gathered by Lombardy Regional Agency for Environmental Protection (ARPA Lombardia) was divided in pre-policy data (from 2014 to 2018) and in the early-stage policy assessment (focusing on eight months of data after the policy implementation). Several factors were considered in the evaluation of models, such as weather conditions, socio-economic factors and pollutants’ concentration in neighboring cities. Despite the fact that the results showed an average reduction of concentrations after the policy implementation, this study highlighted that these changes could be due to other factors than the policy implementation, taking into account that the evaluated short time window may not reflect the citizens’ adaptation to the new rules. Moreover, a negative impact on air quality of some specific areas was found which may be explained by the congestion of public transport buses (promoted by the restrictions) and by the change of the traffic temporal dynamics (since the aforementioned restriction is limited to business hours). Overall, this study characterized the initial phase of the policy implementation, where future assessments should be done to assess its impact when fully implemented, and, additionally, hourly data should also be considered to understand the hourly traffic dynamics and their impact on the local air quality.

A study conducted in Setúbal (Portugal) provided a ten year evaluation (2003–2012) of local air quality trends (focusing on PM_10_, PM_2.5_, O_3_, NO, NO_2_ and NO_x_) and identified potential pollution sources of particulate matter, using a source apportionment technique, namely Positive Matrix Factorization [6]. Overall, air quality improved over the years with a decreasing trend of air pollutant concentrations, with the exception of O_3_. However, despite this improvement, levels of PM_10_, O_3_ and nitrogen oxides still do not fully comply with the requirements of European legislation and the guideline values of World Health Organization (WHO). This study identified the main anthropogenic sources contributing to local PM levels as traffic, industry and wood burning, highlighting the need to address the sources by specific mitigation measures in order to minimize their impact on the local air quality.

Taking into account that road dust resuspension is a current air quality management challenge in Europe, a study conducted in Viana do Castelo (Portugal) focused on this issue and characterized road dust samples collected in suburban and urban streets, defined PM_10_ emissions factors for different road types and identified the main anthropogenic contributions to them [7]. Estimated PM_10_ emission factors ranged from 49 mg∙veh^−1^∙km^−1^ (asphalt) to 330 mg∙veh^−1^∙km^−1^ (cobble stone). Road dusts in urban streets revealed the contribution from traffic emissions (regarding the levels of Cu, Zn and Pb), while in suburban streets, the contribution from agriculture practices was highlighted by As levels in the finest road dust fraction. Hazard quotients were also assessed and a probability of induction of non-carcinogenic adverse health effects in children was found due to ingestion of Zr, while As in the suburban street was found to represent a human health risk of 1.58 × 10^−4^, which reveals the need for adoption of local mitigation measures.

Besides chemical pollutants, air pollution also includes atmospheric bioaerosols, such as bacteria and yeasts. To understand how human hazard to these microorganisms changed over time, their temporal evaluation (focusing on a temporal gap of 10 years) was assessed in the South Western Siberia [8]. Bioaerosol samples obtained in 2006–2008 and 2012–2016 were characterized (by means of growth, morphological and biochemical properties) and the indices of hazards for both bacteria and yeasts were calculated. Overall, hazard to humans of culturable microorganisms in the atmospheric aerosol has not changed significantly over these 10 years (trends are undistinguishable from zero with a confidence level of more than 95%) despite a noticeable decrease in the average annual number of culturable microorganisms per cubic meter (6–10 times for the 10 year period).

Particulate matter (PM) pollution is a common environmental problem in urban areas and, in particular, in urban areas with fast development. In order to understand how the economic growth, urbanization and industrialization affect PM levels, a study targeted the specific case of Liaoning Province (China) where it evaluated PM levels and socio-economic indicators during a period of 16 years (from 2000 to 2015) [9]. Applying statistical tools, such as Granger causality test, it was shown that, in terms of the causal interactions, economic activities, industrialization and urbanization processes all showed positive long-term impacts on changes of PM_2.5_ levels. In terms of contributions, industrialization contributed the most to the variability of PM_2.5_ levels in studied sixteen years, followed by economic growth. These outcomes highlight the need for policy makers to explore more targeted policies to address the regional air pollution issue considering the local development characteristics.

## 4. Indoor Pollution Sources

Indoor micro-environments are the environments where humans spend most of their daily time. Therefore, it is important to understand the different mechanisms that can increase indoor concentration levels (from outdoor infiltration and human activities to specific emissions from products—such as candles—or materials used in within buildings) in order to identify the best measures to decrease the exposure associated to them.

Smoking is a worldwide concern, both regarding direct or indirect health issues and also regarding its contribution to the deterioration of indoor air quality. In the last years, the use of electronic cigarettes (e-cigarettes) and heat-not-burn tobacco (HNBT) has been widely adopted as popular nicotine delivery systems (NDS), mainly among adult demographics. A study aimed to understand the effect on indoor air quality by traditional tobacco cigarettes (TCs) and new smoking alternatives, focusing in a multi-pollutant approach (particulate matter, black carbon—BC, carbon monoxide—CO—and carbon dioxide—CO_2_) in two real life scenarios: home and car [10]. Results showed that particle emissions between the different NDS were significantly different, with TCs corresponding to higher PM10 and ultrafine particle concentrations and HNBT corresponding to the lowest levels registered. Considering that black carbon and CO are released by combustion processes, significantly lower levels were found for e-cigarettes and HNBT since no combustion occurs during their use. However, despite the fact that the new generation of cigarettes results to substantially lower levels of air pollutants compared to TCs, their use still constitutes a source of indoor air pollutants, which should be tackled.

Several actions can be taken in order to reduce indoor air pollutants. For instance, in some countries, such as South Korea, filter-type air purifiers are used to eliminate fine particulate matter from indoor environments. A study assessed the performance of this type of solution to reduce particulate matter levels in a person’s breathing zone, according to two approaches: a movable air purifier responding to the movement of persons in the room or a fixed air purifier [11]. The results showed a decrease of 51 μg/m^3^ and 68 μg/m^3^ from the implementation of these two strategies, respectively.

## 5. Health Impacts of Human Exposure to Air Pollution

Understanding the health impacts of air pollution by means of statistical models is an important step to provide to the scientific community, policy makers and citizens in general the knowledge about the real burden of human exposure to air pollutants. This valuable information is, certainly, the foundation for promoting behavior change and public acceptance to the implementation of measures to tackle the human exposure. Therefore, improving such type of assessment models is very important in order to provide the most accurate and reliable information. Considering only outdoor concentration of air pollutants as a surrogate for total population exposure and neglecting micro-environments where people spend most of their time, may lead to biased and under-evaluated outcomes.

A study focused on this issue on the Greater London Area (UK) [12], including different micro-environments where people spend time at (based on time-activity and housing stock data), identified a misclassification of 1174–1541 mean predicted mortalities attributable to PM_2.5_ exposure in the studied area when only outdoor concentration was considered. Indoor exposure to PM_2.5_ was found to be the largest contributor to total population exposure concentrations accounting for 83%, followed by the exposure in the London Underground with 15%, despite the time spent on it being only 0.4%. Overall, this study confirmed that increasing the models’ complexity and incorporating relevant micro-environments regarding the potential human exposure can significantly reduce the misclassification of health burden assessments.

The use of statistical models can also be employed to explore potential associations between exposure to air pollutants and health outcomes to highlight potential causal relationships. A study conducted in Wuhan city (China) evaluated the time-series of air pollutants (PM_2.5_, PM_10_, SO_2_, NO_2_, CO and O_3_) and focused on the tuberculosis’ incidence in order to identify its potential association with short-term exposure to air pollution, based on kriged data and single and multi-pollutant models [13]. Single pollutant models showed that an increase of 10 μg/m^3^ in concentrations of PM_2.5_, PM_10_ and O_3_ promoted an increase of the associated tuberculosis risk, while in the multi-pollutant model, only PM_2.5_ showed a statistically significant effect on tuberculosis incidence. Despite the complexity of the mechanism linking air pollution and tuberculosis incidence, this study provides insights on potential associations, which should be explored in future research.

A cross-sectional study conducted in Selangor (Malaysia) focused on assessing the allergy and lung inflammation promoted by exposure to different indoor pollutants (PM_2.5_, PM_10_, NO_2_ and CO_2_) in scholar environments, based on marker expression on eosinophils and neutrophils [14]. A total of 96 students from eight suburban and urban schools answered to standardized questionnaires and their fractional exhaled nitric oxide (FeNO) was evaluated, along with allergic skin prick test and sputum induction (which was later analysed for the expression of CD11b, CD35, CD63 and CD66b on eosinophils and neutrophils). By using chemometric and regression analysis, it was found that exposure to PM_2.5_ and NO_2_ was likely associated with the degranulation of eosinophils and neutrophils, following the activation mechanisms that led to the inflammatory reactions.

Adverse birth outcomes (stillbirth, preterm births and low birth weight) due to exposure to household air pollution from unclean cooking fuel in Nigeria were also assessed in a study using Bayesian models, taking into account geographic variability [15]. Using data obtained in a national cross-sectional survey, unclean fuel was the primary source of cooking for 89.3% of the women and the risk of stillbirth was significantly higher for mothers using this type of cooking fuel. It was also found that mothers who attained at least primary education had reduced risk of stillbirth, while for the increasing age of the mother the risk of stillbirth increased. Geographical differences were also found with mothers living in the Northern states having a significantly higher risk of adverse births outcomes.

## References

[1] Schraufnagel D.E., Balmes J.R., Cowl C.T., De Matteis S., Jung S.H., Mortimer K., Perez-Padilla R., Rice M.B., Riojas-Rodriguez H., Sood A. (2019). Air Pollution and Noncommunicable Diseases: A Review by the Forum of International Respiratory Societies’ Environmental Committee, Part 1: The Damaging Effects of Air Pollution. Chest.

[2] Agrawaal H., Jones C., Thompson J.E. (2020). Personal Exposure Estimates via Portable and Wireless Sensing and Reporting of Particulate Pollution. Int. J. Environ. Res. Public Health.

[3] Arano K.A.G., Sun S., Ordieres-Mere J., Gong B. (2019). The Use of the Internet of Things for Estimating Personal Pollution Exposure. Int. J. Environ. Res. Public Health.

[4] Dong D., Xu X., Xu W., Xie J. (2019). The Relationship Between the Actual Level of Air Pollution and Residents’ Concern about Air Pollution: Evidence from Shanghai, China. Int. J. Environ. Res. Public Health.

[5] Maranzano P., Fassò A., Pelagatti M., Mudelsee M. (2020). Statistical Modeling of the Early-Stage Impact of a New Traffic Policy in Milan, Italy. Int. J. Environ. Res. Public Health.

[6] Silva A.V., Oliveira C.M., Canha N., Miranda A.I., Almeida S.M. (2020). Long-Term Assessment of Air Quality and Identification of Aerosol Sources at Setúbal, Portugal. Int. J. Environ. Res. Public Health.

[7] Candeias C., Vicente E., Tomé M., Rocha F., Ávila P., Célia A. (2020). Geochemical, Mineralogical and Morphological Characterisation of Road Dust and Associated Health Risks. Int. J. Environ. Res. Public Health.

[8] Safatov A., Andreeva I., Buryak G., Ohlopkova O., Olkin S., Puchkova L., Reznikova I., Solovyanova N., Belan B., Panchenko M. (2020). How Has the Hazard to Humans of Microorganisms Found in Atmospheric Aerosol in the South of Western Siberia Changed over 10 Years?. Int. J. Environ. Res. Public Health.

[9] Shi T., Hu Y., Liu M., Li C., Zhang C., Liu C. (2020). How Do Economic Growth, Urbanization, and Industrialization Affect Fine Particulate Matter Concentrations? An Assessment in Liaoning Province, China. Int. J. Environ. Res. Public Health.

[10] Savdie J., Canha N., Buitrago N., Almeida S.M. (2020). Passive Exposure to Pollutants from a New Generation of Cigarettes in Real Life Scenarios. Int. J. Environ. Res. Public Health.

[11] Park H., Park S., Seo J. (2020). Evaluation on Air Purifier’s Performance in Reducing the Concentration of Fine Particulate Matter for Occupants according to its Operation Methods. Int. J. Environ. Res. Public Health.

[12] Kazakos V., Luo Z., Ewart I. (2020). Quantifying the Health Burden Misclassification from the Use of Different PM2.5 Exposure Tier Models: A Case Study of London. Int. J. Environ. Res. Public Health.

[13] Huang S., Xiang H., Yang W., Zhu Z., Tian L., Deng S., Zhang T., Lu Y., Liu F., Li X. (2020). Short-Term Effect of Air Pollution on Tuberculosis Based on Kriged Data: A Time-Series Analysis. Int. J. Environ. Res. Public Health.

[14] Mohd Isa K.N., Hashim Z., Jalaludin J., Lung Than L.T., Hashim J.H. (2020). The Effects of Indoor Pollutants Exposure on Allergy and Lung Inflammation: An Activation State of Neutrophils and Eosinophils in Sputum. Int. J. Environ. Res. Public Health.

[15] Roberman J., Emeto T.I., Adegboye O.A. (2021). Adverse Birth Outcomes Due to Exposure to Household Air Pollution from Unclean Cooking Fuel among Women of Reproductive Age in Nigeria. Int. J. Environ. Res. Public Health.

